# The Healing Through Ongoing Psychological Empowerment Telehealth Intervention With Two Spirit, Transgender, and Nonbinary Clients of Color in the United States: Open Clinical Trial Feasibility and Implementation Analysis

**DOI:** 10.2196/64477

**Published:** 2025-05-12

**Authors:** Stephanie Lynne Budge, Elliot Aaron Tebbe, Joonwoo Lee, Sergio Domínguez Jr, Em Matsuno, Louis Lindley

**Affiliations:** 1 Department of Counseling Psychology, University of Wisconsin-Madison, 1000 Bascom Mall, 305 Education Building, Madison, WI, 53706, United States, 1 6082633753; 2 University of Wisconsin-Madison, Madison, WI, United States; 3 School of Counseling and Counseling Psychology, Arizona State University, Tempe, AZ, United States

**Keywords:** Black, Indigenous, people of color, transgender, nonbinary, radical healing, internalized transnegativity, open clinical trial, psychotherapeutic, lack of competence, cultural, humility, psychotherapy, therapist, HOPE, Healing Through Ongoing Psychological Empowerment, gender identity, intervention, content analysis, treatment, medication, mental health

## Abstract

**Background:**

There is a notable lack of psychotherapeutic services tailored to the needs of Two Spirit, transgender, and nonbinary (2STNB) people of color; research indicates that 2STNB clients who are people of color report a lack of competence and cultural humility on the part of their therapists.

**Objective:**

The purpose of this study was to report the feasibility and acceptability of the Healing Through Ongoing Psychological Empowerment (HOPE) teletherapy intervention using deductive content analysis.

**Methods:**

We used an open clinical trial design (testing one intervention without a comparison group) to test the feasibility and acceptability of the HOPE intervention. At baseline, 51 clients were enrolled in the open clinical trial, with 49 2STNB clients who are people of color starting and completing the HOPE intervention. Clients were recruited primarily from social media and therapist waitlists. Clients completed up to 15 free face-to-face telehealth psychotherapy sessions that were provided by nine 2STNB therapists who are people of color. Feasibility and acceptability interviews were conducted prior to the intervention, immediately following the intervention, and at 6 months after completing the intervention.

**Results:**

The HOPE intervention demonstrated high feasibility and acceptability, specifically regarding data collection, psychometric adequacy, interventionist recruitment or training or retention, delivery of the intervention, acceptability of the intervention to clients, and client engagement with the intervention.

**Conclusions:**

These findings propose HOPE as a potentially feasible, culturally specific therapeutic approach for the 2STNB community who are people of color. Future randomized controlled trials comparing HOPE to existing evidence-based treatments are needed.

## Introduction

Psychotherapy serves as an effective method for addressing mental health concerns for Two Spirit, transgender, and nonbinary (2STNB) clients who are people of color. However, 2STNB clients who are people of color experience numerous barriers to accessing competent and affirming psychotherapy services [1], with clients reporting various types of negative experiences with mental health providers, including gender- and race-based microaggressions [2], a general lack of competency [3,4], as well as gatekeeping dynamics when seeking access to medical gender-affirming care [5]. Participants in prior research have described how these negative experiences have undermined the therapeutic relationship, exacerbated distress, and at times led to early termination of therapy [2,6,7]. While much of the existing research has documented these negative experiences in therapy, some studies have found that when barriers to accessing mental health care and oppression are addressed, and clients receive competent and affirming care, psychotherapy effectively reduces mental health symptoms and promotes healing [8,9]. In a recent psychotherapy randomized controlled trial with 19 transgender and nonbinary adults, researchers found that transgender and nonbinary adults readily participated in therapy when barriers to access were reduced (eg, financial barriers), and when therapists were trained to address the harmful impact of identity-based stigma and related stressors [8]. Although the study demonstrated that therapy improved clients’ psychological well-being, and participants reported a strong working alliance with their therapist and overall satisfaction with the therapy, many also reported a desire to work with a transgender or nonbinary therapist. This request could not be accommodated in a trial that only included cisgender therapists.

In their review of race and gender identity dynamics between therapists and clients, Erby and White emphasized [6] the necessity of using an intersectional lens to address layers of systematic oppression within the therapeutic dynamic. Dominguez et al [10] highlighted the importance of using the radical healing framework in therapy to address the racial-gendered experiences of 2STNB clients who are people of color. The authors also stress the need to focus on unpacking the internalization of racial-gendered stigma. The psychological framework for radical healing (PRH) incorporates elements such as critical consciousness, cultural authenticity and self-knowledge, collectivism, radical hope, and cultivating strength and resistance for clients who are people of color [11,12]. The PRH specifically notes that the term “radical” derives from collective resistance that was deemed radical by those who were engaged in oppressive practices. Adames et al [11] defined radical as “a critical attitude or ideology that promotes the idea that complete change is necessary to reduce social problems”; therefore, radical healing in the psychotherapy context involves supporting clients in realizing their full potential in a context where they have experienced gendered and racialized oppression. While the PRH is robust in its conceptualization of healing for people of color, further clarity regarding gendered dimensions of healing could benefit clinicians. In contrast, Israel et al [13] developed an intervention to reduce internalized stigma for transgender and nonbinary people, but this approach lacked a racialized perspective on stigma. The literature clearly indicates a need for an approach that integrates intersectional understandings of both race and gender for 2STNB clients who are people of color.

In this study, we used community-based participatory research (CBPR) methods to design a psychotherapy study aimed to address some of the structural barriers that many 2STNB adults who are people of color face when seeking mental health care. Our CBPR process began at the study’s conception and has continued through its completion and the dissemination of study findings, both in the scholarly literature and within the community. This approach is particularly important against the backdrop of historical and contemporary harms inflicted on 2STNB and people of color by both researchers and mental health professionals [14,15]. CBPR methods can effectively increase transparency of study goals and methods, improve study design to maximize participant experience, and build trust between researchers and community members [16]. Guided by our CBPR framework and processes, this psychotherapy trial implemented four main strategies for providing psychotherapy to 2STNB clients who are people of color: (1) we employed exclusively 2STNB therapists who are people of color to provide psychotherapy to 2STNB clients who are people of color; (2) all therapy sessions were provided at no cost to clients, reducing socioeconomic barriers; (3) by using telehealth, we reduced geographic barriers; and (4) all therapists received specialized training prior to beginning the study to increase their competence in effectively addressing the mental health needs of 2STNB clients who are people of color.

Our main research question for this study included: do results from the open clinical trial demonstrate the feasibility and acceptability of the Healing Through Ongoing Psychological Empowerment (HOPE) intervention? We used the framework of Teresi et al [17] to evaluate the feasibility and acceptability of our trial design and intervention implementation [17]. Specifically, we evaluated whether participants could adequately engage with the data collection protocols; their perceptions of the measures and psychometric adequacy of the measures; whether therapists could be recruited, trained, and retained; whether therapists provided the therapy as intended; and the overall acceptability of therapy to the clients. Our analytic approach for determining feasibility and acceptability is detailed below.

## Methods

### Participants

For this study, our target sample size for the open clinical trial design was 50 participants, each paired with one of the 10 therapists hired for the study (with therapists seeing 5 clients each). Following a screening call for study eligibility, 51 clients were enrolled, and 49 began and completed the psychotherapy intervention. The sample size was determined based on pilot clinical sample size recommendations [18]. All participants were located in the United States. The mean age of participants was 28.35 (SD 6.38; range 21‐52) years. Participants identified their gender, race, and ethnicity using both forced-choice and open-response formats. The open-ended responses showed greater variability and multiple identities, which were used to contextualize participant quotes. Regarding responses to the forced-choice format, 43% (n=22) of the sample were Black or African American, 33% (n=17) were Asian or Asian American, 28% (n=14) were Latine or Latinx or Hispanic (non-White), 12% (n=6) were American Indian or Native American or Indigenous, and 2% (n=1) were Native Hawaiian or Pacific Islander (note: participants could note more than 1 identity; 14% (n=7) also listed a White identity alongside a people of color identity). Regarding forced-choice gender, most clients noted a nonbinary identity (n=39, 77%), while 13% (n=7) identified as transmen, and 10% (n=5) identified as transwomen..

### Therapists

The training provided to therapists prior to participants enrolling in the study integrated approaches for addressing internalized transnegativity [13,19,20] into the PRH [11,12]. The design, development, and implementation of this training are described in greater detail elsewhere [21]. In total, 9 of the 10 intended outpatient community therapists were trained for this clinical trial, as 1 was unable to attend the required training due to health issues. Therapists’ racial or ethnic identities were Asian or Asian American (n=2, 22%), Black or African American (n=3, 33%), Latine or Latinx or Hispanic (n=1, 11%), and multiracial (Indigenous or Latine, White or Latine, and Black or Latine; n=3, 33%). Therapists most often endorsed a nonbinary identity (n=6, 67%), and 3 (33%) identified as women. Therapists were licensed to provide therapy in all but 10 states throughout the United States. More detailed information on therapists’ backgrounds and demographic information are presented in Multimedia Appendix 1.

### Procedure

Procedures of this study were developed using a CBPR model. Specifically, a community advisory board (CAB) was established, consisting of 2STNB community members who are people of color and representatives from organizations serving 2STNB community members who are people of color. The CAB was integrally involved at each stage, providing critical feedback and guidance on various aspects of the study, including participant and therapist recruitment and retention, survey measures and interview protocols, and therapist training. This collaborative process was crucial for shaping the study design to maximize feasibility and acceptability for both 2STNB clients and therapists who are people of color. Furthermore, maintaining transparency throughout the process and ensuring open communication with both clients and therapists helped mitigate mistrust and uncertainty.

Participants were recruited within the United States and lived throughout 8 of 9 census regions (the only region not represented was East South Central [Mississippi, Alabama, Tennessee, and Kentucky]). Clients were recruited via study therapists’ waitlists, social media, community organization listservs, and referrals from other community therapists in states where the study therapists were licensed. The inclusion criteria for clients were (1) being 18 years or older, (2) identifying as 2STNB or having sex assigned at birth different from their current gender, (3) fluency in English or Spanish, and (4) living in a US state where the therapists were licensed. Exclusion criteria included (1) the presence of psychiatric symptoms requiring inpatient treatment (eg, active psychosis) and (2) ongoing individual psychotherapy outside of the study. We kept the inclusion and exclusion criteria minimal to enhance the generalizability of our findings.

Once clients were screened and determined eligible for the study, they scheduled a 2-hour baseline session with study staff via a web-based platform. During this session, study procedures were discussed, informed consent was obtained, and a qualitative interview and baseline surveys were completed. Clients could choose to engage in data collection sessions either in Spanish or English. Additionally, if clients were matched with a bilingual therapist, they could choose to engage in psychotherapy in either Spanish or English. Throughout the study, participants completed qualitative interviews and surveys at 3 time points: prior to starting therapy, immediately following the 15-session limit, and 6 months after the trial ended. These time points were chosen to evaluate initial engagement, immediate outcomes, and longer-term effects of the intervention, providing comprehensive data on the feasibility and acceptability of the study design and therapeutic approach. As part of our CBPR process, we initially consulted with our CAB. The CAB recommended that we present all measures and qualitative questions to focus groups of 2STNB clients who are people of color to determine if items or questions are acceptable, appropriate, and relevant. We conducted 5 focus group meetings, during which we received important feedback on our qualitative interview questions. This feedback was incorporated into our interview protocol.

### Training for Therapists

The HOPE intervention was developed to integrate PRH in psychotherapy for 2STNB clients who are people of color. Our intervention is grounded in the tenets of the PRH [11,12], and a web-based intervention aimed at reducing internalized transnegativity [13]. Initially, the training developed for this study was piloted with 100 therapists through a program evaluation study [21]. Based on feedback from this pilot, the training was adapted for the 9 therapists for this study. All therapists received 16 hours of training and participated in 12 monthly group supervisions throughout the trial. The 2 consultation group leaders were also 2STNB people of color.

### Intervention

Detailed information about the intervention is included in the paper that demonstrates the pilot testing of the training [21]. The intervention incorporates the main tenants of the PRH [11,12] and also from a previously tested internalized transnegativity intervention [13]. The primary components of the PRH that were included in the intervention include critical consciousness, strength and resistance, emotional and social support, cultural authenticity and self-knowledge, and radical hope. The primary components of the internalized transnegativity intervention include opposing stereotypes, recognizing and rebuffing negative messages, strengthening the dismissal of negative messages, and improving identity affirmation. As this was not a manualized treatment, there is not an intervention manual.

### Ethical Considerations

This study was approved by the Institutional Review Board (2021‐1133) at the University of Wisconsin-Madison and was also registered at ClinicalTrials.gov (NCT05140174). Clients provided verbal consent and also electronically signed consent forms that were approved by the institutional review board. As part of our process of protecting the privacy of clients, all qualitative interviews were uploaded to a secure server and transcribed by a team member who deidentified all transcripts. All transcripts were stored on a secure drive and were coded using the secure drive. Clients were compensated US $100 at baseline and postintervention and US $125 at follow-up for their time spent filling out measures and engaging in the interviews. Recruitment began in January 2022. Participants were offered 15 sessions of therapy free of charge, which could be scheduled flexibly (eg, weekly and biweekly) until December 31, 2022.

### Instrument: Feasibility and Acceptability Questions

Clients were asked a series of questions at baseline, postintervention, and 6-month follow-up. These questions covered topics such as feasibility, acceptability, previous therapy experiences, therapy goals within the study, perceptions of the psychotherapy interventions for the study, and experiences of change from therapy. Each interview lasted approximately 60 minutes at each time point. For this paper, we will focus solely on the interview questions related to feasibility or acceptability. At baseline, participants were asked how they heard about the study, why they wanted to participate, any factors contributing to uncertainties about participation, and anticipated barriers to participation. At postintervention and follow-up, participants were asked about their overall experiences in the study, accessibility of telehealth, ease of scheduling sessions, completing measures, perceptions of study procedures, and their openness to participate in the study again if given the opportunity.

### Data Analyses: Qualitative Data Analysis

All qualitative data for the feasibility and acceptability analyses were coded using deductive content analysis following transcription [22]. According to Elo and Kyngäs [22], there are several steps to qualitative data analysis, including the preparation phase, selecting the unit of analysis, and making sense to the data as a whole. In deductive content analysis, the coder develops a structured analysis matrix, codes the data according to categories, and then conducts hypothesis testing. Finally, the coder reports the analysis process and the results, which then leads to the interpretation of the analysis. For this study, the first author (SLB) developed a categorization matrix based on feasibility or acceptability definitions and coded the data accordingly, following the procedures outlined by Elo and Kyngäs [22].

## Results

### Measure Implementation

**Figure 1.**
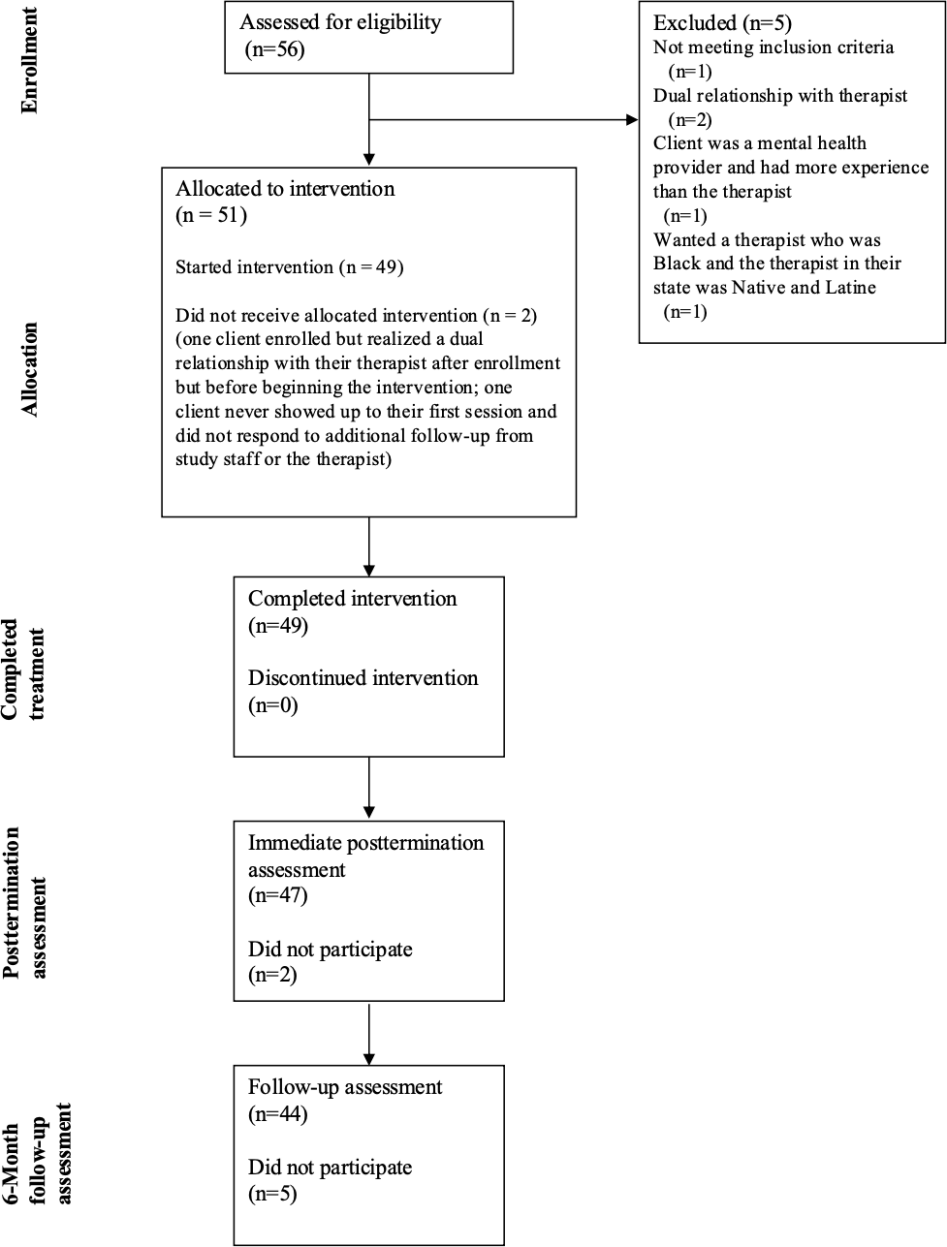
CONSORT (Consolidated Standards of Reporting Trials) diagram: enrollment, allocation, completed treatment, and follow-up assessments for the Healing Through Ongoing Psychological Empowerment intervention.

We conceptualized feasibility based on the guidelines of Teresi et al [17] for reporting pilot and feasibility studies. The first guideline includes assessing if participants could comply with data collection protocols. Clients who participated at each of the 3 main data collection points (baseline: n=51, postintervention: n=47, and 6-month follow-up: n=44) completed all measures and participated in qualitative interviews, demonstrating the feasibility of the main data collection time points (Table 1 and Figure 1). Additionally, we aimed to generate quantitative data at each of the psychotherapy sessions (ie, the Working Alliance Inventory [WAI-C] [23] and the Outcome Questionnaire-45 [24]). Therapists were instructed to remind participants to complete the presession measures before starting each session; however, not all clients filled out the presession surveys. At the postinterview, 10 of 47 (21%) participants indicated that their most frequent criticism of the study was that clients did not like filling out the surveys prior to the sessions, for example, when Alex (all names provided for quotes are pseudonyms; 29 years, Black, trans nonbinary client) was asked about any changes they would make to study procedures, they said: “Not taking the surveys before (laughs) each session um that was a little difficult to do beforehand especially because the timing that [therapist’s name] and I generally would just kinda roll out of bed and show up.” Frankie (26 years, Indigenous or Black, nonbinary trans client) said at the postinterview about the presession surveys: “I’m glad I don’t have to do that no more!” At 6-month follow-up, the criticism about the surveys was less of an issue, but 5 of 44 (11%) clients did indicate that they would have appreciated more reminders for surveys.

**Table 1.** Participant demographic variables prior to starting the Healing Through Ongoing Psychological Empowerment intervention^a^.

	Values
Age (years), mean (SD)	28.35 (6.38)
Annual individual income (US $), mean (SD)	11,491 (18,384)
Race or ethnicity, n (%)	
American Indian or Native American or Indigenous	6 (12)
Asian or Asian American	17 (33)
Black or African American	22 (43)
Latine or Latinx or Hispanic	14 (28)
Native Hawaiian or Pacific Islander	1 (2)
Gender, n (%)	
Nonbinary	39 (77)
Transwoman	5 (10)
Transman	7 (13)

a The baseline sample included 51 clients. Participants could list more than 1 race or ethnicity; therefore, percentages add up to more than 100%. A total of 7 (14%) participants also noted a White identity along with a people of color identity.

Regarding the conceptual and psychometric adequacy of the measures, we assessed whether the measures were acceptable, appropriate, and relevant to the clients in this study. Following completion of the study, 44 of 47 (94%) participants indicated that the measures were acceptable, appropriate, and relevant in the postinterview and 6-month follow-up interview, although some participants offered feedback common to survey research, such as wanting to answer “in-between” a numerical response, or provided a more in-depth explanation for some survey items. At the postinterview, 3 of 47 (6%) clients mentioned that they did not like the questions from the WAI-C and found them challenging to fill out.

### Intervention Implementation

For intervention implementation, we adhered to the framework established by Teresi et al [17] for assessing feasibility. Teresi et al [17] defined feasibility around 4 key aspects. The first aspect includes whether interventionists can be recruited, trained, and retained. We initially aimed to hire 10 community therapists. However, 1 therapist who was initially hired was unable to participate in the training and was therefore not included as a therapist in the study. The remaining 9 therapists were successfully trained and retained. However, during client recruitment, 1 of the 9 therapists was dealing with illness and grief and requested not to take on additional clients. In response, we increased the number of clients assigned to the other therapists in the study while ensuring equitable distribution and maintaining balance.

The second aspect of intervention implementation feasibility includes whether interventionists can deliver the intervention as intended. Therapists were asked about their ability to incorporate radical healing and internalized stigma interventions during the study. All therapists (100%) indicated that they used techniques from the training with each client in the study. When clients were asked about the incorporation of these techniques, 45 of 49 (92%) clients from the postintervention and follow-up indicated that their therapists used radical healing techniques in therapy. For internalized stigma techniques, 42 of 47 (90%) clients from the postintervention and follow-up indicated that their therapists incorporated these techniques into therapy (note: 1 client was not asked about this, resulting in missing data for both questions). Interestingly, 2 clients indicated at the 6-month follow-up that they were using tools from therapy to help reduce internalized stigma, despite saying their therapists did not use specific techniques to reduce internalized stigma at the postinterview.

The third aspect of feasibility for the intervention implementation includes whether the treatment conditions are acceptable to participants and interventionists. At postintervention, all but one participant indicated that they would participate in the study again and found the intervention useful; one participant—Rain (36 years, Black or Afro-American, gender-free, gender-empty, ungendered person)—who indicated they would not participate again stated:

I think I might be leaning towards a “maybe not”? Just because I’m thinking about the different kind of therapy that I’m thinking about moving into next, which would be like a more music therapy–based kind of thing. I have no idea where that would fit into how y’all are thinking about therapy but then also the options of people that you had as therapists were really limited for where I am in the world. So, I think that also would sway me towards a no.

At the same time, this participant indicated that they experienced “so much positive change” because of the intervention and that “it just felt like a very simple, radical healing ... it just felt like this way to offer a simple invitation to living more fully.” At the 6-month follow-up, 44 of 47 (94%) clients indicated they would participate again if they could, while 3 clients indicated that they were “50/50” or “not sure or probably” about participating again. Of these 3 participants, one shared that they felt as though they were in a better place following the completion of the study and would not want to take a spot from someone else who might benefit from the intervention. Despite improvement through the study, another client said that they would prefer to be able to choose who their therapist was rather than being assigned. Finally, the third client felt that they were “only able to scratch the surface” and would have wanted more sessions.

Finally, the last component of feasibility for intervention implementation focuses on adherence and engagement with the intervention. Adherence was not formally assessed in this study, as the therapists operated within a broader framework rather than following a strict protocol. This precluded the need for measuring strict adherence. Regarding engagement, clients were offered up to 15 sessions of psychotherapy but were informed that they did not need to use all of the sessions if they felt it was unnecessary. This approach aimed to assess the feasibility of a 15-session intervention. Of the 49 clients who began the intervention, no clients dropped out of the study, resulting in a 100% retention rate. Session attendance ranged from 7 to 15 sessions (mean 13.88, SD 2.14; median 15, IQR 14-15), with postassessments conducted after the client’s final session. The vast majority of clients completed all 15 sessions (n=32, 65%). Only 5 clients completed 10 or fewer sessions, primarily due to issues with the clients’ schedules. The client who completed the fewest sessions moved out of the country but was able to have a formal termination session with their therapist and complete postintervention and follow-up study sessions.

## Discussion

### Principal Findings

The primary aim of this study was to determine the feasibility and acceptability of the HOPE teletherapy intervention. All components assessed in this study, following the guidelines of Teresi et al [17] for pilot studies, demonstrated both feasibility and acceptability. For the intervention component of the study, we achieved a 100% retention rate; all 49 clients who started the intervention also completed it, though there were varying levels of session attendance. This retention rate is notably higher than the rates reported in meta-analyses of clinical trials, which typically range from 16% to 28% [24-29]. To date, there are no clinical trials that are directly comparable to this one for assessing retention rates. However, researchers indicate that attention to racial and ethnic identity components in interventions reduces attrition in clinical trials [30]. The only other published psychotherapy clinical trial that has focused on 2STNB clients also demonstrated high retention of clients (ie, 95.74%) [8]. We hypothesize that the high retention rate in this open trial was likely due to a combination of factors: our study’s CBPR method and process, which strengthened the design for implementation with 2STNB populations who are people of color; the provision of free, readily accessible outpatient therapy (ie, telehealth); and the flexible scheduling of the 15 sessions over an extended period.

In addition to the completion of the intervention, all but one client indicated at postintervention that they would participate in the study again. The large majority of clients were enthusiastic about the study, with several making unprompted remarks that it was the best research involvement they had experienced. This mirrors findings from a previous randomized clinical trial with 2STNB clients [8]. Besides the reasons listed earlier for high retention, clients appreciated the compensation structure for the 3 waves of data collection and the provision of free outpatient psychotherapy for the duration of the study. Additionally, clients felt that, in addition to benefiting from the intervention individually, they were contributing to scientific efforts to improve mental health interventions for future 2STNB clients who are people of color. This aligns with the critical consciousness component of the PRH, emphasizing the importance of contributing to bettering the experiences of community members [11,12].

Although the feasibility and acceptability data were strong in this study, the least feasible component was the session-by-session surveys (Outcome Questionnaire-45 and WAI-C) that clients were asked to fill out. Many clients indicated that they did not like taking the surveys because they felt the questions were rote or found it difficult to assign a number to their experiences. Studies show that clients in clinical trials rate their satisfaction highest when convenience is high [30,31], and it appears that completing surveys before each session was likely the least convenient aspect of the study design.

### Limitations and Future Research

The present findings should be interpreted with the study’s limitations in mind. A significant limitation of our pilot design is the absence of a control group, which limits our ability to conclusively determine whether the HOPE intervention is more effective than other treatments in improving mental health and reducing internalized stigma among 2STNB individuals who are people of color. Future research should involve a randomized controlled trial that compares the HOPE intervention with either treatment as usual or an additional intervention that has demonstrated efficacy. This future study will be crucial for disseminating evidence-based therapies tailored to the needs of historically marginalized 2STNB individuals who are people of color and can also determine the dose needed for optimal therapy to occur. In addition, this study’s demographic composition, predominantly people located in the United States and nonbinary populations with a female sex at birth, indicates a need for future research to engage a more diverse array of participants. Specifically, the notable underrepresentation of transwomen of color highlights an important gap. Future studies should aim to better understand how to effectively reach and support the particularly underrepresented group within the broader community.

### Conclusions

In this open clinical trial, we developed and implemented the HOPE intervention, the first psychotherapy intervention specifically developed for people of color who are 2STNB, based on PRH and an intervention aimed at reducing internalized transnegativity. We found broad enthusiasm, acceptability, and feasibility for the implementation of the HOPE intervention, specifically regarding data collection, psychometric adequacy, interventionist recruitment or training or retention, delivery of the intervention, acceptability of the intervention to clients, and client engagement with the intervention. Piloting the HOPE intervention is an exciting first step to determining effective interventions for 2STNB clients who are people of color and seeking psychotherapy that addresses stigma and oppression.

## Supplementary material

10.2196/64477 Multimedia Appendix 1 Feasibility or acceptability interview protocol questions.

## Abbreviations

2STNB: Two Spirit, transgender, and nonbinary
CAB: community advisory board
CBPR: community-based participatory research
HOPE: Healing Through Ongoing Psychological Empowerment
PRH: psychological framework for radical healing
WAI-C: Working Alliance Inventory
