# Supplementary material for: The Healing Through Ongoing Psychological Empowerment Telehealth Intervention With Two Spirit, Transgender, and Nonbinary Clients of Color in the United States: Open Clinical Trial Feasibility and Implementation Analysis
Source: JMIR Form Res. 2025 May 12;9:e64477. doi: 10.2196/64477 (PMC12088614; doi:10.2196/64477)
Supplement: Multimedia Appendix 1 [file formative-v9-e64477-s001.docx]

Baseline:

1. How did you hear about this study?
2. Can you tell us about why you’re interested in participating in this study?
3. Was there anything about study that made you unsure if you wanted to participate?
4. What kinds of barriers do you think will exist for you to finish the study? (e.g., having a quiet and private place to talk, Internet or technology, family obligations, etc.)?
5. What are you looking forward to with participating in this study?

Post-Study and Six-Month Follow-Up:

1. Now that you are finished participating in the study, what are your thoughts on your overall experience of the study?
2. From start to finish, what study procedures do you wish had been different?
3. What parts of the study made it easier for you to be in therapy?
4. What parts of the study made it harder for you to be in therapy?
5. If you had the opportunity, would you participate in this study again? If yes or no, explain why? If not sure, have them expand upon answer.
6. How would you like to receive updates about the study (for example, what would be helpful materials to receive so you know about the result?)
